# Prognostic factors in patients with metastatic spine tumors derived from lung cancer—a novel scoring system for predicting life expectancy

**DOI:** 10.1186/s12957-018-1439-x

**Published:** 2018-07-05

**Authors:** Hiroshi Uei, Yasuaki Tokuhashi

**Affiliations:** Department of Orthopaedic Surgery, Nihon University School of Medicine, 30-1 Oyaguchi-kamicho, Itabashi-ku, Tokyo, 173-8610 Japan

**Keywords:** Metastatic spine tumor, Lung cancer, Molecule-targeting drug, Prognostic evaluation system, Scoring system, Treatment modality

## Abstract

**Background:**

Recently, molecule-targeting and bone-modifying agents have improved the treatment outcomes of lung cancer-derived metastatic spine tumors. Therefore, the prognostic factors for such tumors were examined, and novel scoring systems for predicting the life expectancy of patients with such tumors were proposed.

**Methods:**

In 207 patients with lung cancer-derived metastatic spine tumors (surgery 49; conservative therapy 158), we retrospectively examined the factors that influenced the post-treatment survival time (age, sex, the affected site, pathology, general condition, the number of extraspinal bone metastases, the number of spinal metastases, the presence/absence of major internal organ metastasis, paralysis state, the total Tokuhashi score, the serum alkaline phosphatase level, the serum carcinoembryonic antigen level, molecule-targeting drug treatment, and bone-modifying agent treatment). Based on the results, we devised novel scoring systems for predicting the prognosis of such patients.

**Results:**

Univariate analyses showed that the pathology of the primary lung tumor, the patient’s general condition and paralysis state, and the presence/absence of molecule-targeting drug treatment significantly influenced survival. We performed a Cox regression analysis of these four factors and developed criteria for a novel scoring system based on the patient’s general condition and paralysis state, which exhibited significance in the regression analysis. A retrospective review indicated that the consistency rate between predicted life expectancy and actual survival was 67.3%. When criteria based on the four factors that exhibited significance in the univariate analyses were adopted, the consistency rate was 76.2%.

**Conclusion:**

The patient’s general condition and paralysis state, the pathology of the primary lung tumor, and molecule-targeting drug treatment influenced survival among patients with lung cancer-derived metastatic spine tumors. Novel scoring systems based on these four factors were proposed.

## Background

Bone metastasis from lung cancer is detected in approximately 30 to 40% of patients with advanced non-small cell lung cancer, who exhibit a median survival time of < 1 year. A retrospective study involving patients with non-small cell lung cancer in Japan showed that the incidence of bone metastasis during the follow-up period was 30.4%. The most frequent site of bone metastasis was the spine [1].

Metastatic spine tumors derived from lung cancer exhibit rapid progression, leading to an unfavorable prognosis. In fact, they are the most difficult to treat of all metastatic spine tumors [2]. In many patients, paralysis progresses rapidly, and the most appropriate treatment must be determined promptly [2–6]. Recently, molecule-targeting and bone-modifying agents (BMA) have improved the treatment outcomes of lung cancer and lung cancer-derived spinal metastasis [7–11].

Since 1990, the optimal treatment for metastatic spine tumors has been selected based on prognostic predictions obtained using a revised version of the Tokuhashi score (Table 1) [12, 13]. In lung cancer patients, the maximum total Tokuhashi score is 10 points, and it is difficult to predict a life expectancy of ≥ 1 year using the present criteria. We used to use the Tokuhashi scoring system, which we developed, to determine the optimal treatment strategy for lung cancer patients with metastatic spine tumors. The ability of this system to predict the prognosis of patients with spinal metastasis has been examined in many previous studies around the world [14–19]. However, recently, as patients with lung cancer-derived metastatic spine tumors have started to survive longer, greater discrepancies have been seen between life expectancy predictions obtained using the Tokuhashi score and actual post-treatment survival times. Therefore, a new prognostic evaluation system is required for determining the optimal treatment options for such patients, including whether spinal surgery should be performed. In this study, we examined the prognostic factors for lung cancer-derived metastatic spine tumors and proposed novel scoring systems for predicting the prognoses of patients with such tumors.

**Table 1:** A revised Tokuhashi score [13]

Predictive factor	Score (points)
General condition	
Poor (KPS 10–40%)	0
Moderate (KPS 50–70%)	1
Good (KPS 80–100%)	2
Number of extraspinal bone metastases foci	
≥ 3	0
1–2	1
0	2
Number of metastases in the vertebral body	
≥ 3	0
2	1
1	2
Metastases to the major internal organs	
Unremovable	0
Removable	1
No metastases	2
Primary site of the cancer	
Lung, osteosarcoma, stomach, bladder, esophagus, pancreas	0
Liver, gallbladder, unidentified	1
Others	2
Kidney, uterus	3
Rectum	4
Thyroid, prostate, breast, carcinoid tumor	5
Spinal cord palsy	
Complete (Frankel A, B)	0
Incomplete (Frankel C, D)	1
None (Frankel E)	2
Total points	Predicted prognosis
0–8	< 6 months
9–11	≥ 6 months
12–15	≥ 1 year

*KPS* indicates Karnofsky’ s performance status

## Methods

### Subjects

The subjects were 207 patients with lung cancer-derived metastatic spine tumors (surgery 49; conservative therapy 158) who visited our department after 2000.

The inclusion criteria were as follows: the patient had a lung cancer-derived metastatic spine tumor and was symptomatic; the patient complained of back pain and pain and/or paralysis of the extremities; the treatment was selected in collaboration with oncologists and radiotherapists based on an evaluation of the pathology of the lung cancer, its sensitivity to adjuvant treatment, and the patient’s general condition and expected survival time; the decision-making process and any treatments were administered promptly and could be adjusted appropriately in urgent cases; and the date of the patient’s death was known.

The exclusion criteria were as follows: (1) patients who were administered the main constituent of the initial treatment for the spinal metastasis at another institution, (2) patients who dropped out of the follow-up process, and (3) patients whose date of death could not be confirmed.

The surgical indications were as follows: (1) pain and/or paralysis caused by spinal instability, (2) pain and/or paralysis due to spinal cord invasion by the tumor, and (3) long-term local control in patients with localized lesions and a life expectancy of at least 1 year. The surgical procedures consisted of posterior stabilization with decompression in 27 patients, posterior stabilization without decompression in 10 patients, posterior decompression alone in 2 patients, anterior stabilization with decompression in 8 patients, anteroposterior stabilization with decompression in 1 patient, and total en bloc spondylectomy in 1 patient. Of the 158 patients who underwent conservative therapy, symptomatic therapy alone was administered to 37 patients. Adjuvant therapy was administered to the remaining 121 patients and most of the patients that underwent surgery. Finally, chemotherapy (excluding molecule-targeting drugs), radiotherapy, molecule-targeting drugs, and BMA were administered to 96, 102, 59, and 143 patients, respectively. Cisplatin and carboplatin were usually used for the chemotherapy. In the radiotherapy, 20–30 Gy (in 2 to 3 Gy fractions) radiation were administered. The administered molecule-targeting drugs included gefitinib, erlotinib, bevacizumab, axitinib, crizotinib, and afatinib. In principle, 250 mg oral gefitinib were administered daily for adenocarcinoma if a mutation was detected in the epidermal growth factor receptor gene. If the gefitinib became insufficient, 150 mg erlotinib or 40 mg oral afatinib were administered daily. 500 mg oral crizotinib were administered daily for adenocarcinoma when the ALK gene was detected.

The administered BMA included zoledronic acid, a bisphosphonate, and denosumab, an anti-receptor activator of nuclear factor kappa-B ligand (RANKL) antibody. 4 mg/month intravenous zoledronate or 120 mg/month subcutaneous denosumab were administered where possible. In principle, the BMA were administered as soon as spinal metastasis was detected.

Based on the patients’ status and wishes, multiple treatment methods were employed where possible.

### Methods

This was a retrospective and mono-institutional study. We examined the factors that influenced the post-treatment survival time, including age, sex, the affected site, the pathology of the primary lung tumor, the patient’s general condition, the number of extraspinal bone metastases, the number of spinal metastases, the presence/absence of main organ metastasis (especially the liver metastasis), the patient’s paralysis state, the total Tokuhashi score, the alkaline phosphatase (ALP) level, the carcinoembryonic antigen (CEA) level, the presence/absence of molecule-targeting drug treatment, and the presence/absence of BMA treatment. The post-treatment survival time was defined as the period from the start of treatment for spinal metastasis until death. In other words, the post-treatment survival time included the treatment period.

Based on these results, we devised novel scoring systems for predicting the prognosis of patients with lung cancer-derived metastatic spine tumors by combining the factors that had a significant influence on patient prognosis.

The study protocol was approved by the institutional ethics committee of Nihon University Itabashi Hospital (approval number RK-11209-8).

### Statistical analysis

To investigate the factors that influenced the post-treatment survival time, univariate analyses were performed using the *t* test, Welch’s method, or analysis of variance (ANOVA). A multivariate analysis involving the factors that exhibited significance in the univariate analyses was conducted using Cox’s regression analysis. We used the StatMate V® software (Atoms Co., Tokyo, Japan) for all statistical analyses.

## Results

### Post-treatment survival time

The mean post-treatment survival time of the 207 patients with lung cancer-derived metastatic spine tumors was 8.26±12.15 months (range 0.2–114 months).

### Univariate analyses of the factors that influenced the post-treatment survival time

#### Sex

The subjects consisted of 136 males and 71 females, who exhibited mean survival times of 7.1±8.7 and 10.8±16.6 months, respectively (*p* = 0.0803).

#### Age

The mean survival times of the subjects aged ≤ 69 years (*n* = 117) and ≥ 70 years (*n* = 90) were 8.9±14.0 and 7.8±9.2 months, respectively (*p* = 0.8002).

#### The affected site (symptom level)

Cervical, thoracic, and lumbosacral lesions were seen in 27, 119, and 61 patients, respectively. In these 3 groups, the mean survival time was 5.9±7.3, 10.3±15.8, and 5.9±7.3 months, respectively (*p* = 0.1603).

#### Pathology of the primary lung cancer

We investigated the cases of 177 patients in whom a definitive pathological diagnosis was finally made (120, 24, 24, and 9 patients were diagnosed with adenocarcinoma, small cell carcinoma, squamous cell carcinoma, and others, respectively). The mean survival times of the adenocarcinoma and non-adenocarcinoma patients were 10.0±14.0 and 6.4±7.6 months, respectively (*p* = 0.0263).

#### Tokuhashi score parameters

We also examined the prognostic significance of the factors included in each category of the Tokuhashi score (Table 1). The patients’ general condition was evaluated using Karnofsky’s performance status (KPS) at the start of treatment for spinal metastasis, i.e., they were divided into poor (KPS 10–40%) (*n* = 22), moderate (KPS: 50–70%), (*n* = 30), and good (KPS 80–100%) (*n* = 126) groups. The mean survival times of the poor, moderate, and good groups were 2.1±2.4, 5.4±6.1, and 11.1±14.3 months, respectively. There were significant differences between the survival times of the poor and good groups (*p* = 0.0019) and between those of the moderate and good groups (*p* = 0.0321).

In addition, the subjects were divided into three groups based on the number of extraspinal bone metastases that they possessed, i.e., into those with ≥ 3 (*n* = 99), 1–2 (*n* = 17), and 0 (*n* = 40) extraspinal bone metastases. The mean survival times of these three groups were 10.3±14.7, 11.1±15.3, and 11.0±19.7 months, respectively. The difference among them was not significant (*p* = 0.9604).

Furthermore, we examined how the number of spinal metastases influenced the mean survival time. As a result, we found that it was 8.1±9.4, 13.9±23.4, and 6.3±7.3 months in the patients with ≥ 3 (*n* = 121), 2 (*n* = 29), and 1 (*n* = 57) spinal metastases, respectively. The difference among them was not significant (*p* = 0.1388).

As for the effect of the presence/absence of major internal organ metastasis, the mean survival time was 10.5±16.7 months in the 132 patients with major internal organ metastasis and 11.7±12.9 months in the 22 patients without such metastasis (*p* = 0.6994). In addition, the mean survival time was 8.1±9.3 months in the patients with liver metastasis (*n* = 46) and 11.7±12.9 months in those without it (*p* = 0.3543).

Paralysis was evaluated using Frankel’s classification. The mean survival time was 5.2±3.3 months in the patients with complete paralysis (Frankel: A, B), 5.5±7.0 months in those with incomplete paralysis (Frankel C, D), and 11.3±16.7 months in those that were free from paralysis (Frankel E). There was a significant difference between the mean survival times of the incomplete paralysis and paralysis-free groups (*p* = 0.0279).

With respect to the total Tokuhashi score, the mean survival time was 9.1±14.6 months in the patients with scores of ≤ 8 points (life expectancy < 6 months) and 16.7±16.3 months in those with scores of 9 to 10 points (life expectancy ≥ 6 months) (*p* = 0.1542).

#### Laboratory data

The laboratory data obtained at the start of treatment for spinal metastasis were analyzed. The patients with ALP levels of < 400 IU/L (*n* = 85) and ≥ 400 IU/L (*n* = 70) exhibited mean survival times of 13.0±20.5 and 8.8±9.5 months, respectively (*p* = 0.0961). An ALP cut-off level of 400 IU/l was employed based on the finding that an ALP level of ≥ 400 IU/l is a potential indicator of high bone metastasis activity [20].

Furthermore, the patients with CEA levels of < 120 ng/ml (*n* = 111) and ≥ 120 ng/ml (*n* = 36) displayed mean survival times of 10.2±14.9 and 8.9±1 months, respectively (*p* = 0.5189). A CEA cut-off level of 120 ng/ml was employed based on the fact that a CEA level of > 120 ng/ml was reported to be associated with an increased risk of bone metastasis [2].

#### Presence/absence of molecule-targeting drug treatment

Of the various conservative treatments administered to the patients in this study, only molecule-targeting agents and BMA were used as part of a consistent treatment strategy. The other conservative treatments were not used as part of a consistent treatment strategy because their indications or the doctors’ approach to treatment changed over time. Therefore, these other conservative treatments could not be evaluated as prognostic factors. The administered molecule-targeting drugs included gefitinib, erlotinib, bevacizumab, axitinib, crizotinib, and afatinib. In 59 patients, these molecule-targeting drugs were used for ≥ 1 month. In 126 patients, molecule-targeting drug treatment was discontinued within 1 month due to adverse reactions or no such drugs were used.

The former and latter groups exhibited mean survival times of 15.0±21.1 and 7.3±10.4 months, respectively (*p* = 0.0093).

#### Presence/absence of bone-modifying agent treatment

In 143 patients, BMA, such as zoledronic acid or denosumab, were used. In 64 patients, no such drugs were used.

The former and latter groups exhibited mean survival times of 8.2±15.8 and 8.6±10.2 months, respectively (*p* = 0.8650).

### Multivariate analyses of the factors that influence post-treatment survival

The results of the univariate analyses are shown in Table 2. In the univariate analyses, the pathology of the primary lung cancer, the patient’s general condition and paralysis state, and the presence/absence of molecule-targeting drug treatment were found to significantly influence post-treatment survival. Multivariate analysis was performed using Cox’s regression analysis among the 168 patients for whom data on the four abovementioned factors were available.

**Table 2:** Significant differences of survival periods (months)

Factor	*p* value
Sex.	
Male:female	0.0803
Age (years) ≤ 69 vs ≥ 70	0.8002
Symptomatic level Cervical vs thoracic vs lumbosacral	0.1603
Pathology Adenocarcinoma vs non-adenocarcinoma	0.0263*
Tokuhashi score	
General condition	
Poor vs good	0.0019*
Moderate vs good	0.0321*
Number of extraspinal bone metastases foci	0.9604
Number of metastases in the vertebral body	0.1388
Metastases to the major internal organs	0.6994
Paralysis (spinal cord palsy)	0.0279*
Total score ≤ 8 vs 9–10	0.1542
ALP 400 IU/L ≥ or <	0.0961
CEA 120 ng/ml ≥ or <	0.5189
Molecular target drug, use (for ≥ 1 month) or not	0.0093*
Bone-modifying agent, use or not	0.8650

*Significant difference (*p* < 0.05). *ALP* indicates Alkaline phosphatase, *CEA* carcinoembryonic antigen

**Fig. 1:**
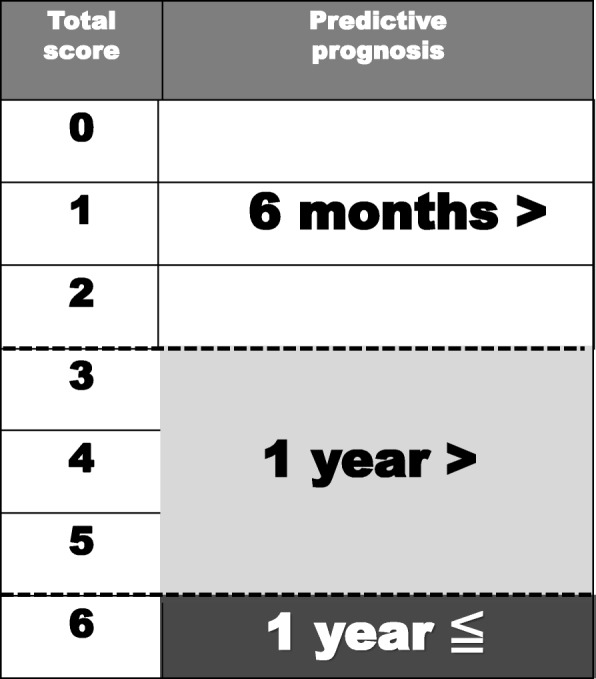
Criteria for predicting the prognosis of patients with lung cancer-derived metastatic spine tumors (maximum total score 6 points). Total scores of 0 to 2 points, 3 to 5 points, and 6 points were indicative of a life expectancy of < 6 months, < 1 year, and ≥ 1 year, respectively

**Fig. 2:**
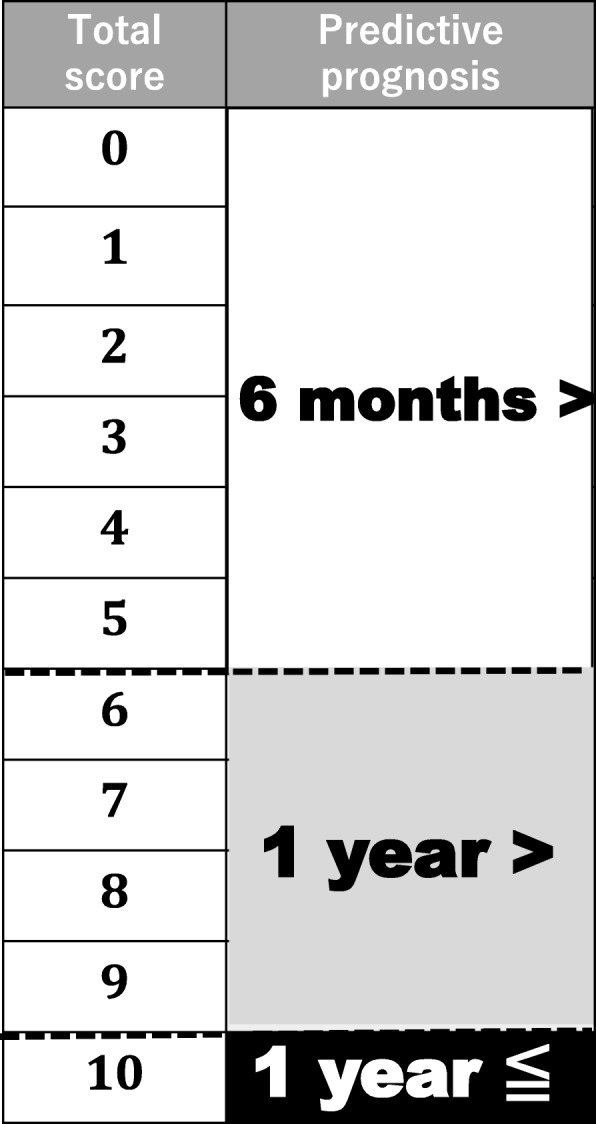
Criteria for predicting the prognosis of patients with lung cancer-derived metastatic spine tumors (maximum total score 10 points). Total scores of 0 to 5 points, 6 to 9 points, and 10 points were indicative of a life expectancy of < 6 months, < 1 year, and ≥ 1 year, respectively

The patient’s general condition and paralysis state were found to have significant effects on survival (Table 3). Based on the hazard ratios for these two factors, we created novel scoring criteria for predicting the prognoses of patients with lung cancer-derived metastatic spine tumors (Table 4, Fig. 1). The maximum total score was 6 points (general condition 4; state of paralysis 2) (Table 4), and total scores of 0 to 2 points, 3 to 5 points, and 6 points indicated a life expectancy of < 6 months, < 1 year, and ≥ 1 year, respectively (Fig. 1). A retrospective review of 168 subjects demonstrated consistency rates of 87.5, 84.4, and 47.5% between the life expectancy predictions obtained using the novel scoring system and survival. The overall consistency rate was 67.3% (Table 5). In addition, we prepared a second scoring system for predicting the prognoses of patients with lung cancer-derived metastatic spine tumors based on the four factors that exhibited significance in the univariate analyses (Table 4, Fig. 2). The criteria for this scoring system consisted of the patient’s general condition (4 points) and paralysis state (2 points), the pathology of the primary lung cancer (2 points), and the presence/absence of molecule-targeting drug treatment (2 points). The maximum total score was 10 points, and total scores of 0 to 5 points, 6 to 9 points, and 10 points indicated a life expectancy of < 6 months, < 1 year, and ≥ 1 year, respectively (Table 4, Fig. 2). A retrospective review of 168 subjects demonstrated consistency rates of 82.6, 77.6, and 64.8% between the life expectancy predictions obtained using the second scoring system and survival. The overall consistency rate was 76.2% (Table 5).

**Table 3:** Multivariate analysis of the prognostic factors affecting survival

Factor	Hazard ratio	95% confidence interval	*p* value
Pathology Adenocarcinoma or not	0.933	0.667–1.306	0.6874
General condition (KPS) 10–40 or 50–70 or 80–100	0.406	0.323–0.510	9.10403E-15*
Paralysis (Frankel grade) A, B or C, D or E	0.738	0.558–0.976	0.0333*
Molecular targeted drug use or not	0.867	0.722–1.041	0.1263

*Mean *p* value was less than 0.05, and considered statistically significant

KPS indicates Karnofsky’ s performance status

**Table 4:** A novel scoring system of predicting life expectancy for the patients with metastatic spine tumor from lung cancer

Predictive factor	Score (points)
General condition	
Poor (KPS 10–40%)	0
Moderate (KPS 50–70%)	2
Good (KPS 80–100%)	4
Paralysis	
Complete (Frankel A, B)	0
Incomplete (Frankel C, D)	1
None (Frankel E)	2
Pathology	
Non-adenocarcinoma	0
Adenocarcinoma	2
Molecular target drug	
No use	0
Use	2

6 points predictive score: general condition + paralysis

10 points predictive score: general condition + paralysis + pathology + molecular target drug

KPS indicates Karnofsky’ s performance status

**Table 5:** 6 and 10 points predictive score vs survival periods (months)

		Survival periods				
6 points predictive score	Score	6 months>	6–12 months	12 months <	Total	Mean ± SD
	0	0	0	0	0	
	1	3	0	0	3	1.50 ± 0.87
	2	18	3	0	21	2.30 ± 3.05
	3	4	1	3	8	8.84 ± 9.41
	4	14	4	1	19	6.29 ± 10.6
	5	26	5	6	37	6.21 ± 7.27
	6	26	16	38	80	15.6 ± 20.0
Total		91	29	48	168	
10 points	0	0	0	0	0	
predictive score	1	1	0	0	1	2.5
	2	6	1	0	7	2.50 ± 3.85
	3	4	0	1	5	5.30 ± 7.71
	4	12	2	1	15	5.84 ± 12.0
	5	15	2	1	18	4.41 ± 5.78
	6	17	5	7	29	9.28 ± 12.4
	7	9	5	4	18	8.51 ± 9.31
	8	16	10	6	32	8.69 ± 11.6
	9	4	0	2	6	7.13 ± 6.24
	10	7	6	24	37	20.6 ± 24.9
Total		91	31	46	168

## Discussion

The prognosis of patients with metastatic spine tumors depends on the type of tumor. The survival times of patients with some types of carcinoma have increased due to advances in treatment, but this is not the case for patients with other types of carcinoma. Lung cancer patients belong to the former group [10]. We previously used a revised version of the Tokuhashi score to predict the prognosis of lung cancer patients [13], but the introduction of molecule-targeting drugs (gefitinib) and BMA from 2002 onwards has markedly improved the prognosis of lung cancer patients. Thus, such patients are surviving for longer than they are predicted to according to the Tokuhashi score. Hessler C et al. also reported that patients with lung cancer-derived spinal metastasis are surviving longer [21].

On the other hand, recent studies have indicated that the median survival time of patients with spinal metastasis from pulmonary adenocarcinoma was 3.5 or 5.2 months [5, 6]. In other words, the survival of patients with spinal metastasis from lung cancer varies, and so the importance of predicting patient prognosis when selecting the optimal treatment strategy for lung cancer-derived spinal metastasis has increased.

Goodwin CR et al. indicated that the factors that influence the prognosis of patients with lung cancer-derived spinal metastasis include age, motor function, the presence/absence of major internal organ metastasis, and the presence/absence of extra-thoracic metastasis [6].

Aydinli U et al. reported that among patients with spinal metastasis, those with squamous cell carcinoma showed the most favorable prognosis followed by adenocarcinoma and small cell carcinoma patients [3]. It is unclear why the adenocarcinoma group exhibited a significantly better prognosis than the non-adenocarcinoma group in our series. Since adenocarcinoma accounted for more than two-thirds of our cases, the subjects were divided into two pathology groups, the adenocarcinoma and non-adenocarcinoma groups. Generally, lung cancer patients with squamous cell carcinoma have a better prognosis than those with adenocarcinoma, and it has been suggested that lung cancer patients with small cell carcinoma have a worse prognosis than those with adenocarcinoma. In the present study, the non-adenocarcinoma group only included a few cases of squamous cancer, whereas it included relatively many cases of small cell cancer. We consider that this might explain why the mean survival time of the adenocarcinoma group was longer than that of the non-adenocarcinoma group. In addition, the fact that molecule-targeting drugs could be administered to the adenocarcinoma group based on genetic screening might have contributed to the marked prognostic improvement associated with the use of molecule-targeting drugs.

Lei M et al. reported that the patient’s preoperative gait, the presence/absence of major internal organ metastasis, and the interval from onset until motor paralysis influenced the prognosis of lung cancer patients with spinal metastasis [22]. In univariate analyses, Park SJ et al. found that the prognostic factors for spinal metastasis derived from non-small cell cancer included the time since the appearance of neurological findings, the response to preoperative chemotherapy, postoperative chemotherapy, performance status, and postoperative gait [5]. The subsequent multivariate analysis demonstrated that the time since the appearance of neurological findings, postoperative chemotherapy, and performance status were significant prognostic factors. According to Sugiura H, being female, a favorable performance status, adenocarcinoma, gefitinib (a molecule-targeting drug) treatment, having a solitary lesion, and the absence of long bone metastasis contributed to a favorable prognosis in lung cancer patients with bone metastasis [23]. Katagiri et al. [11] also reported that molecule-targeting drug treatment influences the prognosis of patients with bone metastasis and reported a scoring system involving these drugs for predicting the prognosis of such patients.

Furthermore, a previous study indicated that ALP and CEA [2] measurements are useful for the auxiliary diagnosis of bone metastasis. However, in our series, these factors did not have a significant influence on survival. The serum level of bone-specific ALP has been reported to be useful for predicting the prognosis of patients with bone metastasis or renal cancer [24, 25], but the level of this ALP isozyme was not assessed in the current study. Therefore, an increased ALP level is not a specific finding of spinal metastasis derived from lung cancer because elevated ALP levels are seen in other diseases. Future studies should examine whether the serum level of bone-specific ALP is a prognostic factor for patients with spinal metastasis from lung cancer.

Finally, a recent study investigated the size of the psoas muscle as a prognostic factor in lung cancer patients with spinal metastasis [26].

Despite the abovementioned findings, it should be noted that none of the examined factors have been definitively proven to influence the prognosis of patients with lung cancer-derived spinal metastasis. In addition, our scoring system was created based on the results of a retrospective study. Thus, an additional study of our 10-point scoring system should be conducted.

As for the limitations of this study, it was a retrospective study (rather than a prospective randomized study). In addition, the subjects were restricted to symptomatic patients with metastatic spine tumors. Regarding laboratory data, the serum level of bone-specific ALP should be evaluated as a prognostic factor in future.

Furthermore, our study included a factor relating to the therapeutic intervention, i.e., the presence/absence of molecule-targeting drug treatment. Unexpected factors, such as treatment discontinuation due to adverse reactions, were also included; however, not all patients were indicated for fixed treatments. Except for molecule-targeting drug or BMA treatment, none of the conservative treatments could be evaluated as prognostic factors. This is also a limitation of the current study, and a further evaluation is necessary once a clear treatment strategy for lung cancer-derived metastatic spine tumors has been established.

According to the standard approach, our prognostic scoring system should only have included the two factors that displayed significance in the multivariate analysis, i.e., the patient’s general condition and paralysis state. However, a retrospective review showed that a scoring system involving a combination of the four factors that exhibited significance in the univariate analyses, i.e., the patient’s general condition and paralysis state, the pathology of the primary lung cancer, and the presence/absence of molecule-targeting drug treatment, was more accurate. However, the efficacy of our 2- and 4-factor scoring systems must be reviewed in a larger number of patients in the future.

## Conclusions

The results of the univariate analyses demonstrated that the patient’s general condition and paralysis state, the pathology of the primary lung tumor, and molecule-targeting drug treatment influenced survival among patients with lung cancer-derived metastatic spine tumors. The novel scoring systems based on these four factors was more accurate than that based on the two factors that exhibited significance in the multivariate analysis. These simple scoring systems are useful for aiding the selection of appropriate treatment modalities for progressive spinal metastasis. Additional clinical studies with larger sample sizes are required to further validate these findings.
